# A Genome-Wide Screen with Nicotinamide to Identify Sirtuin-Dependent Pathways in *Saccharomyces cerevisiae*

**DOI:** 10.1534/g3.115.022244

**Published:** 2015-12-07

**Authors:** John S. Choy, Bayan Qadri, Leah Henry, Kunal Shroff, Olatomiwa Bifarin, Munira A. Basrai

**Affiliations:** * Department of Biology, The Catholic University of America, Washington, D.C. 20064; † Genetics Branch, Center for Cancer Research, National Cancer Institute, Bethesda, Maryland 20892

## Abstract

Sirtuins are evolutionarily conserved NAD-dependent deacetylases that catalyze the cleavage of NAD^+^ into nicotinamide (NAM), which can act as a pan-sirtuin inhibitor in unicellular and multicellular organisms. Sirtuins regulate processes such as transcription, DNA damage repair, chromosome segregation, and longevity extension in yeast and metazoans. The founding member of the evolutionarily conserved sirtuin family, *SIR2*, was first identified in budding yeast. Subsequent studies led to the identification of four yeast *SIR2* homologs *HST1*, *HST2*, *HST3*, and *HST4*. Understanding the downstream physiological consequences of inhibiting sirtuins can be challenging since most studies focus on single or double deletions of sirtuins, and mating defects in *SIR2* deletions hamper genome-wide screens. This represents an important gap in our knowledge of how sirtuins function in highly complex biological processes such as aging, metabolism, and chromosome segregation. In this report, we used a genome-wide screen to explore sirtuin-dependent processes in *Saccharomyces cerevisiae* by identifying deletion mutants that are sensitive to NAM. We identified 55 genes in total, 36 of which have not been previously reported to be dependent on sirtuins. We find that genome stability pathways are particularly vulnerable to loss of sirtuin activity. Here, we provide evidence that defects in sister chromatid cohesion renders cells sensitive to growth in the presence of NAM. The results of our screen provide a broad view of the biological pathways sensitive to inhibition of sirtuins, and advance our understanding of the function of sirtuins and NAD^+^ biology.

Sirtuins are class III NAD-dependent deacetylases that serve key roles in the assembly of repressive chromatin structures, genome integrity, chromosome segregation, and are the targets of caloric restriction-mediated longevity extension in some systems (Aparicio *et al.* 1991; Nasmyth 1982; Rine and Herskowitz 1987; Lin *et al.* 2000; Holmes *et al.* 1997; Tissenbaum and Guarente 2001; Pillus and Rine 1989; Choy *et al.* 2011). Sirtuin-catalyzed deacetylation is coupled with the cleavage of NAD^+^ into nicotinamide (NAM) and 2′O-acetyl ADP-ribose (Figure 1A). Moreover, NAM is an effective pan-sirtuin noncompetitive inhibitor in both single celled eukaryotes and metazoans (Avalos *et al.* 2005; Zhao *et al.* 2004). Thus, the balance between NAD^+^ and NAM levels can modulate the activity of sirtuins and influence a range of biological functions. NAM has been shown to influence tumorigenesis in mice and humans as well as alleviating Alzheimer’s-associated pathologies in mice (Yiasemides *et al.* 2009; Gupta 1999; Gotoh *et al.* 1988; Bryan 1986; Zhang *et al.* 2013; Gong *et al.* 2013; Liu *et al.* 2013). Inhibition of sirtuins is thought to underlie the efficacy of some NAM-based therapies but the precise mechanism of NAM action and the downstream targets of sirtuins remains unclear in many cases. Therefore, elucidation of pathways/genes that are affected by NAM is crucial for understanding pathways that are dependent on sirtuin activity and may help to identify therapeutic targets for sirtuin-related diseases.

**Figure 1:**
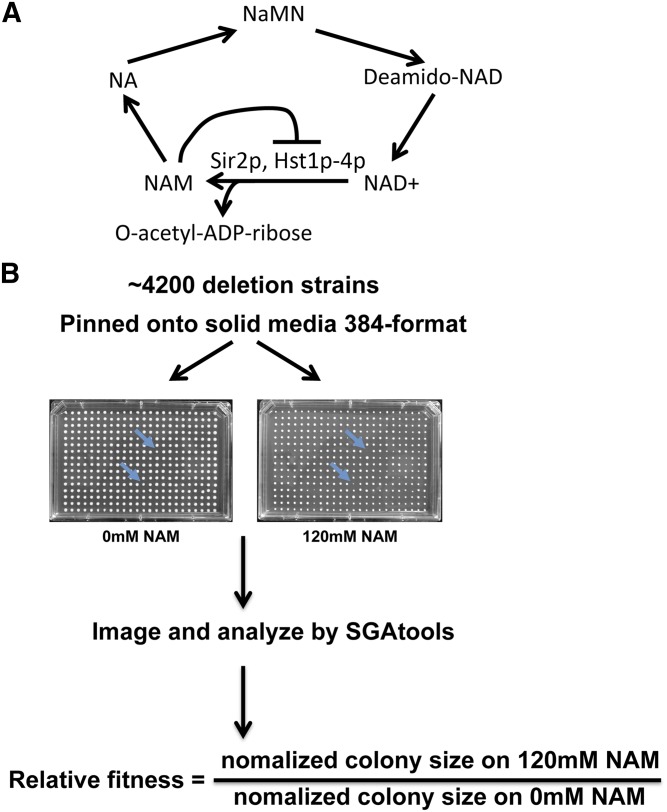
A genome-wide screen for identifying deletion mutants sensitive to NAM. (A) Diagram showing the general pathway utilized by yeast to generate nicotinamide adenine dinucleotide (NAD^+^). Sirtuins use NAD^+^ as a cofactor and generate nicotinamide (NAM) as well as 2′O-acetyl ADP-ribose during a single deacetylation event. NAM can be converted to nicotinic acid (NA), which in turn is used to generate nicotinic acid mononucleotide (NaMN). Next, NaMN is converted to Deamido-NAD, and in turn NAD^+^ is regenerated. (B) Approach used to screen and score deletion mutants that are sensitive to NAM. A collection of ∼4200 yeast deletion mutants were arrayed in 384-format on YPD agar containing 0 or 120 mM NAM. Arrows indicate examples of the fitness defect observed in NAM sensitive mutants. Relative fitness was determined from normalized colony sizes obtained by analysis of plate images using SGAtools.

Budding yeast, which has five sirtuins (*SIR2*, *HST1*, *HST2*, *HST3*, *HST4*), provides an effective model system to study sirtuin biology (Blander and Guarente 2004; Smith *et al.* 2002). Sir2p is the prototypic sirtuin, first discovered in yeast, that regulates chromatin structure by deacetylating key acetylated lysine residues found on histone H3 and H4 (Smith *et al.* 2002). Yeast treated with NAM display defects in transcriptional silencing, hyper-recombination at the rDNA locus, sister chromatid cohesion, and have reduced lifespan (Tripathi *et al.* 2012; Gallo *et al.* 2004; Anderson *et al.* 2003; Thaminy *et al.* 2007). We previously reported that mutants in the yeast centromeric specific histone, *CSE4*, are sensitized to NAM and treatment of wild-type cells with NAM increases the frequency of chromosome loss (Choy *et al.* 2011). Studies have shown that yeast treated with NAM have a reduced replicative lifespan that is associated with hyper-acetylation of histone H3K56 and H4K16, in part through inhibition of Sir2p (Bitterman *et al.* 2002; Hachinohe *et al.* 2011; Choy *et al.* 2011). In addition, assembly of sister-chromatid cohesion and DNA damage repair are promoted by Hst3p- and Hst4p-mediated deacetylation of H3K56, demonstrating shared substrates among yeast sirtuins and the importance of histone acetylation/deacetylation in genome maintenance mechanisms (Maas *et al.* 2006; Celic *et al.* 2006, 2008; Thaminy *et al.* 2007). This redundancy can obfuscate the identification of sirtuin-dependent biological processes using single or double sirtuin deletions. Moreover, genome-wide approaches to investigate the myriad of biological activities using multiple deletions in sirtuins, which include *SIR2*, require a method to bypass the *sir2*Δ mating defect (Rine and Herskowitz 1987; Shore *et al.* 1984; Ivy *et al.* 1986; Liu *et al.* 2010). To circumvent these limitations, we used NAM at a concentration that inhibits all five sirtuins in a genome-wide screen to identify gene deletions that confer sensitivity to NAM. Here, we report the results of our screen and provide novel insights into biological processes that are dependent on sirtuin activity.

## Materials and Methods

### Genome-wide screen to identify gene deletions sensitive to NAM

A *Saccharomyces cerevisiae* library of deletions in ∼4200 nonessential genes in BY4741 was generously provided by the Boone laboratory (Toronto, Canada). A VersArray Colony Arrayer (Bio-Rad, Hercules, CA) equipped with a 384-pinning head was used to array out the library on 15 YPD agar plates, using Omni plates from Nunc, and allowed to grow at 30° for 3 d. Colonies were then pinned onto fresh YPD plates or YPD + 120 mM NAM (N3376 from Sigma-Aldrich, St. Louis, MO) plates, incubated for 3 d at 30°, then imaged with a Nikon digital camera. Images were analyzed by SGAtools as described below. Strains available upon request.

### Quantitative analysis of genome-wide screen

Images of all plates were analyzed using SGAtools (sgatools.ccbr.utoronto.ca) (Wagih *et al.* 2013). Ratios of normalized colony sizes from NAM treated and untreated mutants were used as a measure of sensitivity. Normalized colony sizes were used to determine sensitivity by comparing growth on YPD *vs.* YPD + 120 mM NAM. The screen was performed twice and Table 1 indicates the score obtained for each screen. The raw scores for all mutants from both replicate screens are found in Supporting Information, Table S2 and Table S3.

**Table 1:** Scores for the top 59 mutants from genome-wide screen

ORF	Gene Name	Screen 1	Screen 2	Average Score	Genome Stability*a*
*GOS1*	YHL031C	0.12	0.24	0.18	
*SMI1*	YGR229C	0.31	0.42	0.37	
*SRB2*	YHR041C	0.41	0.41	0.41	**+**
*BUB1*	YGR188C	0.21	0.19	0.20	**+**
*FBP26*	YJL155C	0.09	0.24	0.17	**+**
*MPH1*	YIR002C	0.21	0.26	0.24	**+**
*POL32*	YJR043C	0.24	0.28	0.26	**+**
*LAS21*	YJL062W	0.32	0.41	0.36	
*SWF1*	YDR126W	0.00	0.03	0.02	
*TOP3*	YLR234W	0.34	0.21	0.27	**+**
*VPS53*	YJL029C	0.11	0.17	0.14	**+**
*UBP3*	YER151C	0.39	0.24	0.32	**+**
*YDR455C*	YDR455C	0.31	0.28	0.30	
*HHY1*	YEL059W	0.29	0.33	0.31	
*RAD51*	YER095W	0.15	0.45	0.30	**+**
*BST1*	YFL025C	0.28	0.50	0.39	
*SPF1*	YEL031W	0.33	0.47	0.40	
*RPO41*	YFL036W	0.39	0.43	0.41	
*RPL19B*	YBL027W	0.26	0.20	0.23	
*MRC1*	YCL061C	0.37	0.19	0.28	
*SLX5*	YDL013W	0.37	0.36	0.37	**+**
*PER1*	YCR044C	0.19	0.46	0.32	**+**
*RIC1*	YLR039C	0.33	0.23	0.28	
*SWI6*	YLR182W	0.20	0.33	0.27	
*YJL175W*	YJL175W	0.18	0.49	0.33	
*BUB3*	YOR026W	0.10	0.17	0.13	
*DIA2*	YOR080W	0.32	0.36	0.34	**+**
*PAP2*	YOL115W	0.27	0.32	0.29	**+**
*VAM3*	YOR106W	0.20	0.35	0.27	
*VAM10*	YOR068C	0.17	0.42	0.30	
*SHE4*	YOR035C	0.25	0.40	0.32	
*TLG2*	YOL018C	0.41	0.49	0.45	
*YPT6*	YLR262C	0.22	0.17	0.20	
*ARC1*	YGL105W	0.18	0.29	0.24	
*COG8*	YML071C	0.19	0.20	0.20	**+**
*YMR031W-A*	YMR031W-A	0.13	0.08	0.10	
*YNL171C*	YNL171C	0.37	0.21	0.29	
*YMR166C*	YMR166C	0.14	0.48	0.31	
*COG6*	YNL041C	0.20	0.30	0.25	
*SAC1*	YKL212W	0.00	0.05	0.02	
*CPS1*	YJL172W	0.33	0.50	0.41	
*RPE1*	YJL121C	0.07	0.27	0.17	
*HTZ1*	YOL012C	0.41	0.48	0.44	
*JHD2*	YJR119C	0.31	0.32	0.32	**+**
*DPB3*	YBR278W	0.41	0.42	0.41	
*EAF1*	YDR359C	0.29	0.13	0.21	**+**
*MNN10*	YDR245W	0.12	0.13	0.13	**+**
*PPH3*	YDR075W	0.31	0.19	0.25	
*SPT3*	YDR392W	0.05	0.34	0.20	**+**
*SWI4*	YER111C	0.19	0.18	0.19	
*SNX4*	YJL036W	0.29	0.24	0.26	**+**
*ASC1*	YMR116C	0.14	0.21	0.17	
*COG7*	YGL005C	0.14	0.28	0.21	**+**
*VAM7*	YGL212W	0.00	0.21	0.11	
*DBF2*	YGR092W	0.12	0.46	0.29	**+**
*CKB1*	YGL019W	0.27	0.47	0.37	
*LEA1*	YPL213W	0.40	0.24	0.32	
*VPS1*	YKR001C	0.00	0.00	0.00	
*ERG3*	YLR056W	0.38	0.27	0.33	

a Genome stability (+) indicates that the respective gene has been reported to have function(s) in pathway(s) important for maintaining genome integrity.

### Growth assays to validate results from genome-wide screens

Yeast media and techniques were performed as described (Guthrie and Fink 1991). Yeast strains for growth assays were from the deletion collection (described in *Genome-wide screen*) provided by Dr. Charles Boone or as indicated in Table S6. Cultures of each strain were grown overnight in 96-well plates, serially diluted five-fold, and then 3–4 μl of each dilution was spotted onto agar plates and grown at the indicated temperatures. Typically, plates were imaged after 3–5 d of incubation at the indicated temperatures. Shown are representative spot tests from three independent replicate assays. Yeast strains are described in Table S6.

### Gene ontology mapper

Generic Gene Ontology (GO) Term Mapper (go.princeton.edu/cgi-bin/GOTermMapper) was used to bin the 55 top scoring genes into GO terms (Boyle *et al.* 2004). The results are plotted in Figure 3. GeneMANIA (genemania.org) was used to analyze the reported genetic and physical interactions for the 55 top scoring genes shown in Figure 4 (Zuberi *et al.* 2013).

## Results and Discussion

### Genome-wide screen for mutants that are sensitive to NAM

NAM is a precursor molecule used to synthesize NAD^+^ and an inhibitor of sirtuins both *in vivo* and *in vitro* (Figure 1A) (Kato and Lin 2014). To gain a better understanding of pathways regulated by NAM and sirtuins, we performed a genome-wide screen to identify gene deletion strains that displayed sensitivity to sublethal levels of NAM. We screened a collection of deletions in ∼4200 nonessential genes in 384-format on YPD agar plates containing either no NAM or 120 mM NAM (Figure 1B). The screen was performed twice and plates were incubated at 30° for 2–3 d and then imaged. We used SGAtools to quantify and analyze the colony sizes of strains grown in the absence or presence of NAM (Wagih *et al.* 2013). Ratios of normalized colony sizes between NAM treated and control plates were calculated, and mutants with ratios ≤0.5 were selected as sensitive if they scored similarly in both replicate screens (Table 1, Table S1, Table S2, and Table S3). Based on this criterion, a total of 59 mutants were considered sensitive (Table 1).

**Figure 2:**
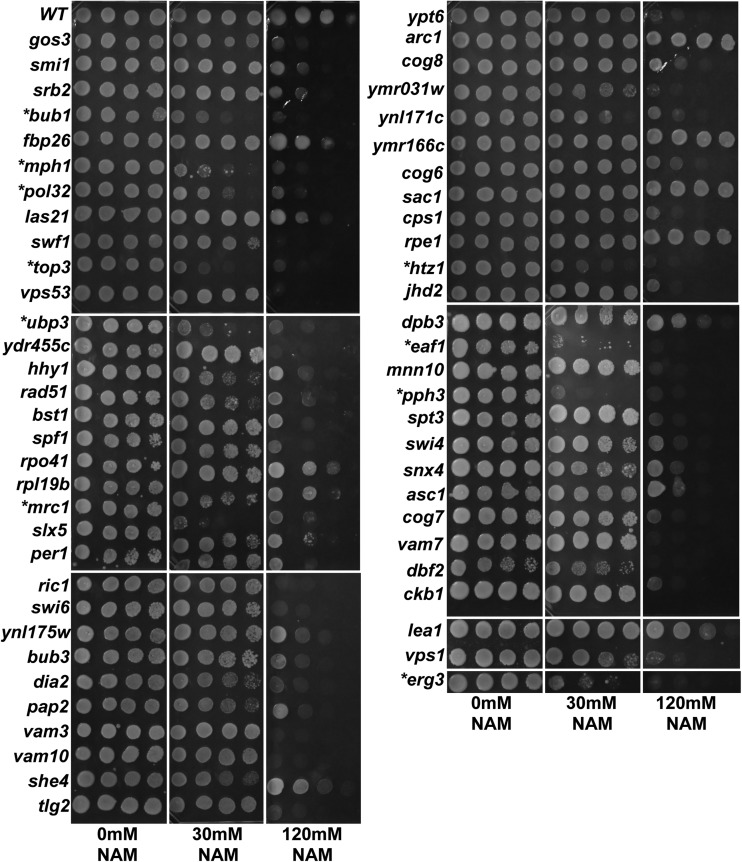
Growth assays for sensitivity of deletion mutants to increasing concentrations of NAM. Nearly 90% of mutants initially identified in the screen show greater sensitivity to 120 mM nicotinamide (NAM) compared to a wild-type control (WT). Scores for the growth in the genome-wide screen for each strain are shown in Table 1. A subset of mutants is considered highly sensitive when there is a loss of viability even at 30 mM NAM. Asterisks indicate the most sensitive mutants. Three biological replicates were done and results were similar for all three experiments. Overnight cultures of each deletion strain were serially diluted five-fold and 3 μl were spotted on indicated media and incubated at 30°C for 2–3 d.

**Figure 3:**
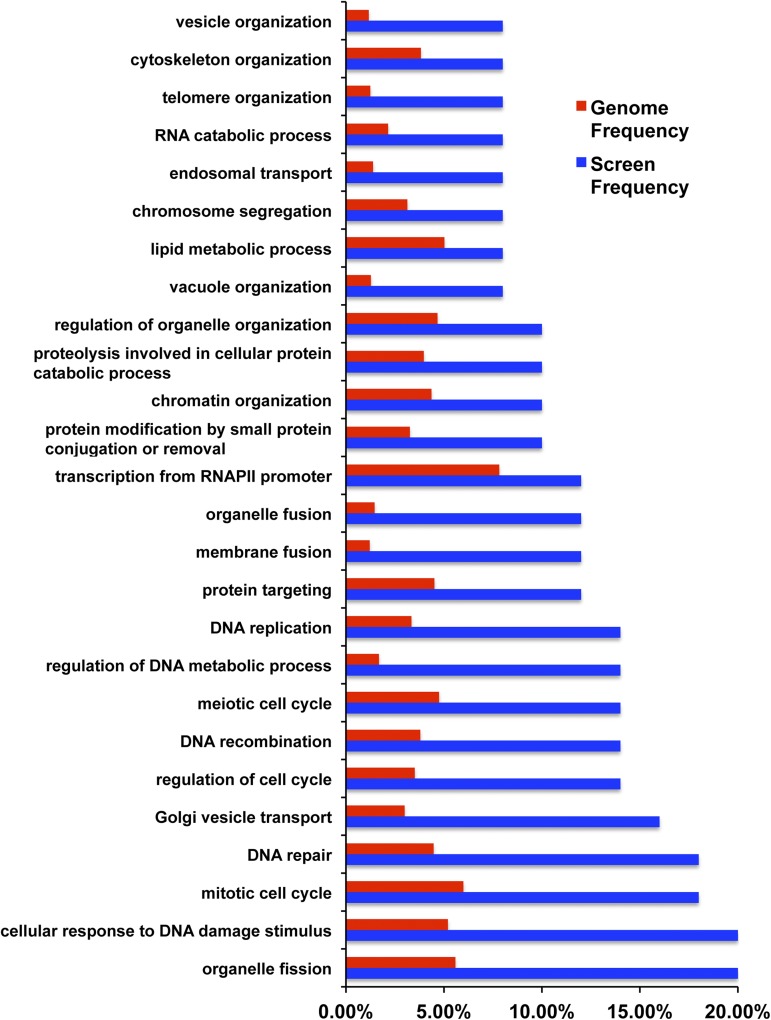
Gene Ontology (GO) Term Mapper indicates a variety of processes affected by nicotinamide (NAM) treatment. The 55 most sensitive deletions are in genes with nuclear functions such as DNA replication and repair, and mitosis. A subset of these deletions is in genes with functions in lipid metabolism and organelle organization. Screen frequency (blue) and genome frequency (red) represent the frequency of observing genes with the respective GO terms on the y-axis. Note that the frequency of occurrence of genes in a given category, except for RNA polymerase II transcription, is several fold higher in our screen compared to the genome.

**Figure 4:**
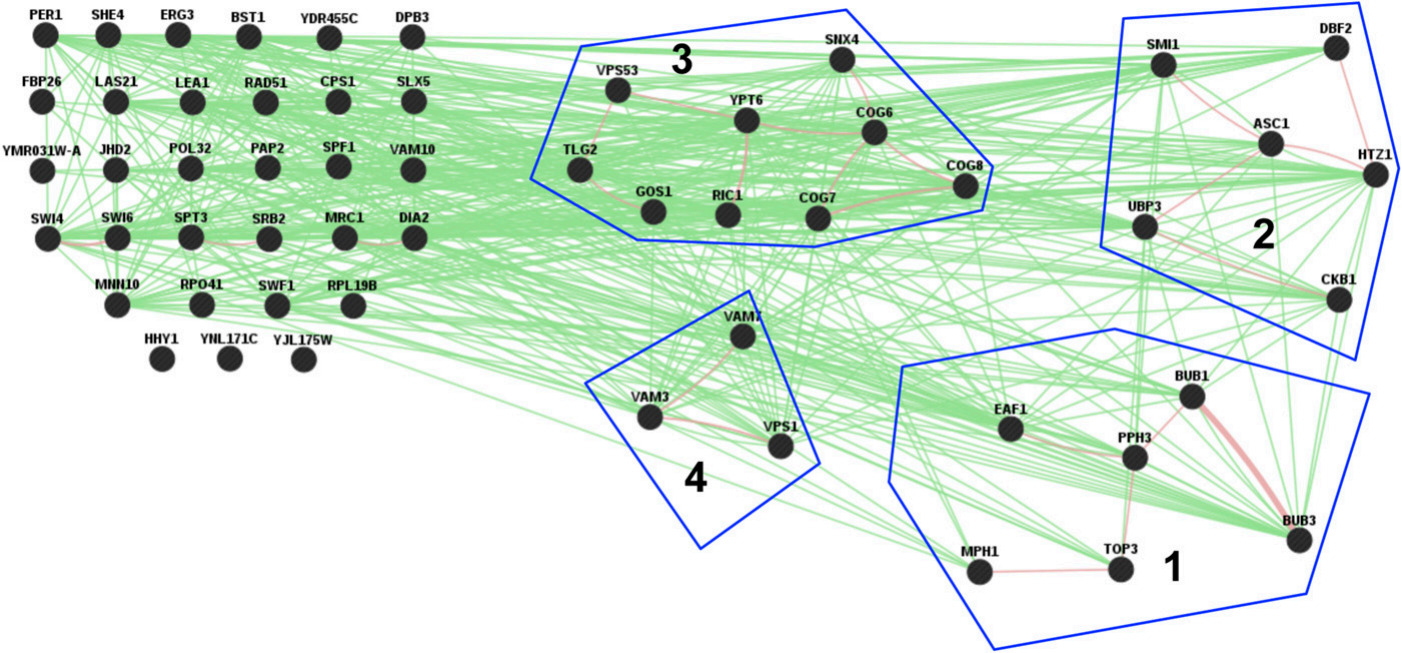
Genetic and physical interactions between the highest scoring 55 genes. Green and pink edges indicate genetic and physical interactions, respectively. Groups 1, 2, 3, and 4 indicate physical interaction networks within a subset of the proteins encoded by their respective genes. Genetic and physical interactions were determined using GeneMANIA to analyze the top 55 scoring genes.

To validate the results of the screen, we performed growth assays using spot tests for each of the 59 deletions. We observed a high rate of true positives in which 55/59 of the mutants tested confirmed their respective sensitivity to 120 mM NAM. Only deletions in *ARC1*, *YMR166C*, *SAC1*, and *RPE1* displayed no compromise in growth on NAM (Figure 2). In addition, we found that 16% of mutants (*UBP3*, *HTZ1*, *MRC1*, *EAF1*, *PPH3*, *BUB1*, *MPH1*, *POL32*, *VPS1*, *ERG3*) displayed marked growth sensitivity even in the presence of much lower concentrations of NAM (30 mM) (Figure 2). Gene Ontology Term Mapper of the 55 sensitive mutants indicated that categories related to mitotic cell cycle, DNA repair, replication, and recombination were highly represented (Figure 3 and Table S5). We note that organelle fission is also highly represented but nearly every gene in that category is known to function in the spindle assembly checkpoint or in DNA damage repair and recombination (Table S5). These results suggest that disruption of pathways that preserve genomic integrity render cells highly vulnerable to excess NAM. In addition, we found that only 19 of the 55 genes we identified have been previously reported to exhibit a negative genetic interaction with any individual sirtuin deletion strain, and nearly 62% (34/55) of genes have human homologs based on YeastMine (Table S4) (Balakrishnan *et al.* 2012). These results suggest that sirtuin-dependent pathways are evolutionarily conserved and may yield critical insights into sirtuin biology in humans. Analysis by GeneMANIA, which provides information on functional association between genes of interest, revealed that over 50% (30/55) of the encoded gene products are reported to have physical interactions with each other, 24 being grouped into one of four physical interaction networks with at least three or more members (Figure 4). Most of the highly sensitive mutants (*UBP3*, *HTZ1*, *EAF1*, *PPH3*, *BUB1*, *MPH1*, *VPS1*) were also found within each of the four physical interaction networks. There are a large number of genetic interactions between these 55 genes that are not part of the physical interaction networks, suggesting that many have overlapping functions (Figure 4). Moreover, a subset of mutants found in interaction network 1 (*BUB1*, *BUB3*, *TOP3*) support a possible role for sirtuins in the regulation of sister chromatid cohesion, which is essential for faithful chromosome segregation.

### Gene deletions for NAM sensitivity form physical interaction networks

Twenty-eight of the 55 genes identified in the NAM screen can be classified into four physical networks, representing either genes required for genome stability and the DNA damage response (networks 1 and 2) or Golgi and vacuolar functions (networks 3 and 4) (Figure 4). Although the genes that comprise networks 3 and 4 have well-established roles in Golgi and vacuolar functions, they nonetheless might have important roles in the DNA damage response pathway. For example, gene deletions in Golgi and vacuolar functions not only exhibit defects in these pathways but also show sensitivity to DNA damaging agents (Costanzo *et al.* 2014; Skrzypek and Hirschman 2011). Furthermore, negative genetic interactions have been reported between deletions in Golgi/vacuolar genes and mutations in genes with functions in genome stability (Costanzo *et al.* 2014; Skrzypek and Hirschman 2011). Thus, all four networks likely have important roles in genome integrity mechanisms.

### Physical interaction network 1: genomic stability

Within this network there are seven genes (*BUB1*, *BUB3*, *PPH3*, *EAF1*, *TOP3*, and *MPH1*) with functions in genomic stability (Figure 4A). Bub1p and Bub3p form a key kinase complex that regulates the spindle assembly checkpoint and centromeric recruitment of the cohesion Sgo1p to ensure faithful chromosome segregation (Hoyt *et al.* 1991; Kawashima *et al.* 2010). Top3p is the catalytic subunit of a trimeric complex that associates with Rmi1p and Sgs1p to form the topoisomerase III complex, which resolves recombination intermediates and plays a role in chromosome cohesion assembly (Lai *et al.* 2007). Eaf1p is a component of the NuA4 histone acetyltransferase complex that acts as a platform where subunits of NuA4 assemble and function in transcription and DNA damage repair (Auger *et al.* 2008). Mph1p encodes a 3′-5′ DNA helicase similar to the human Fanconi anemia group protein that regulates error-free bypass of DNA lesions (Zheng *et al.*, 2011).

### Physical interaction network 2: DNA repair, protein trafficking, and translation

Within this network there are eight genes (*CKB1*, *UBP3*, *ASC1*, *SMI1*, *HTZ1*, *DBF2*) that function in DNA damage repair, protein trafficking, and translation (Figure 4A).

Ckb1p is the regulatory subunit of casein kinase 2, which functions in transcription of RNA PolIII genes that can be activated during DNA damage (Guillemain *et al.* 2007). Ubp3p is an ubiquitin protease with functions in transport between the ER and Golgi and its protein levels increase as a result of replication stress (Cohen *et al.* 2003; Bilsland *et al.* 2007; Tkach *et al.* 2012). Asc1p is the yeast ortholog of RACK1 (receptor for activated protein kinase C1) and is a component of the 40S ribosomal subunit that acts as a translational inhibitor. It also functions as a G-protein β subunit for G alpha protein, Gpa2, and can bind to adenylate cyclase, thereby decreasing cAMP production (Zeller *et al.* 2007; Tkach *et al.* 2012; Coyle *et al.* 2009). Smi1p regulates cell wall synthesis and coordinates its synthesis with cell cycle progression (Martin-Yken *et al.* 2003). HTZ1 encodes a histone H2A variant (H2AZ) that has important functions in transcriptional regulation, while the SWR1 complex mediates exchange of canonical H2A for H2AZ at promoter sites (Mizuguchi *et al.* 2004; Zhang *et al.* 2005). H2AZ has been proposed to play a role in centromeric chromatin and in DNA damage repair (Van *et al.* 2015; Krogan *et al.* 2004). Dbf2p is a kinase that functions in the mitotic exit network and in stress responses (Lee *et al.* 2001).

### Physical interaction network 3: Golgi transport/traffic

This network is comprised of a group of nine genes (*COG6*, *COG7*, *COG8*, *SNX4*, *YPT6*, *RIC1*, *VPS53*, *TLG2*, and *GOS1*) which have functions primarily related to Golgi transport/traffic (Figure 4A). Cog4p, Cog6p, and Cog7p are part of a multi-subunit cytosolic tethering complex that traffics protein to mediate the fusion of vesicles to the Golgi (Kudlyk *et al.* 2013; Loh and Hong 2004). Snx4p is a member of the sorting nexin family that functions in cytoplasm-to-vacuole protein transport and in autophagy (Hettema *et al.* 2003). Ypt6p is a Ras-like GTP binding protein that is required for vesicle fusion with the late Golgi (Luo and Gallwitz 2003). Ric1p is involved with retrograde transport to the *cis*-Golgi and together with Rgp1p acts as a Ypt6p GTP exchange factor (Siniossoglou *et al.* 2000). Vps53p is one of four subunits that comprise the GARP (Golgi-associated retrograde protein) complex that recycles proteins from endosomes to the late Golgi and is involved with DNA damage arrest recovery (Conibear *et al.* 2003). Tlg2p is one subunit of a trimeric complex that mediates fusion of vesicles derived from endosomes with the late Golgi (Abeliovich *et al.* 1998). Gos1p is a v-SNARE protein that functions in Golgi transport (McNew *et al.* 1998).

### Physical interaction network 4: vacuolar trafficking

This is the smallest network composed of three genes (*VPS1*, *VAM3*, *VAM7*) that are critical for vacuolar trafficking (Figure 4A). Vps1p is a dynamin-like GTPase that plays a role in vacuolar sorting, endocytosis, and peroxisome biogenesis (Ekena *et al.* 1993). Vam3p and Vam7p are vacuolar SNARE proteins that function together in vacuolar trafficking (Sato *et al.* 1998).

### Genes encoding proteins that are not members of physical interaction networks 1–4

Twenty-seven of the 55 genes identified in the NAM screen do not fit within the four physical interaction networks (Figure 4). Based on information from the *Saccharomyces* Genome Database (SGD), five of the 27 genes are predicted to be dubious ORFs as they overlap with verified ORFs. Nonetheless, many genetic interactions are present between all 27 genes, between each other, and within the other 28 genes (Figure 4). Importantly, nearly a third of these genes (*ERG3*, *PAP2*, *SWI4*, *SWI6*, *DPB3*, *DIA2*, *MRC1*, *SLX5*, *RAD51*, *POL32*) have functions in DNA replication and repair (Table 1). This further supports the possibility that NAM treatment affects genome integrity. The remaining 16 verified genes encode proteins with functions in organelle trafficking/transport/morphogenesis (*BST1*, *CPS1*, *SPF1*, *SWF1*, *HHY1*, *SHE4*, *VAM10*), anabolic processes such as lipid and GPI synthesis (*LAS21*, *PER1*), gluconeogenesis (*FBP26*, *MNN10*), translation/RNA processing (*LEA1*, *RPL19B*), and transcription (*SPT3*, *SRB2*, *JHD2*) (Table 1 and Table S1). As indicated by SGD, there are genetic interactions between these genes and the DNA replication/repair genes suggesting that they may be operating directly or indirectly in genome integrity mechanisms.

The five deletions that occur in dubious ORFs are *YDR455C*, *YMR031W-A*, *YNL171C*, *YJL175W*, and *YPR050C*. Information for each of the five dubious ORFs was obtained from SGD. It is likely that replacement of the dubious ORF by *kanMX* disrupts the overlapping verified ORF. Deletion of *YDR455C* removes the first 196 bp of *NHX1*, which encodes a Na^+^/H+ and K+/H+ exchanger. *YMR031W-A* overlaps with the first 34 bp of *EIS1*, which encodes a component of the eisosome. *YNL171C* overlaps with 153 bp of the very end of *APC1*, encoding the largest subunit of the anaphase-promoting complex. *YJL175W* overlaps with 481 bp of the beginning of *SWI3* transcription factor. *YPR050C* nearly overlaps completely with *MAK3* (beginning at 7 bp of the 5′-end and ending at 124 bp before the end of *MAK3*), the catalytic subunit of N-terminal acetyltransferase. The data from large-scale studies indicates that deletions in all of these dubious ORFs, except *YDR455C*, confer sensitivity to DNA damage agents. It remains unknown if the phenotypes associated with deletion of these dubious ORFs are due to a loss-of-function in the overlapping ORFs.

### Loss of cohesion function in BUB1 and BUB3 confers NAM sensitivity

**Figure 5:**
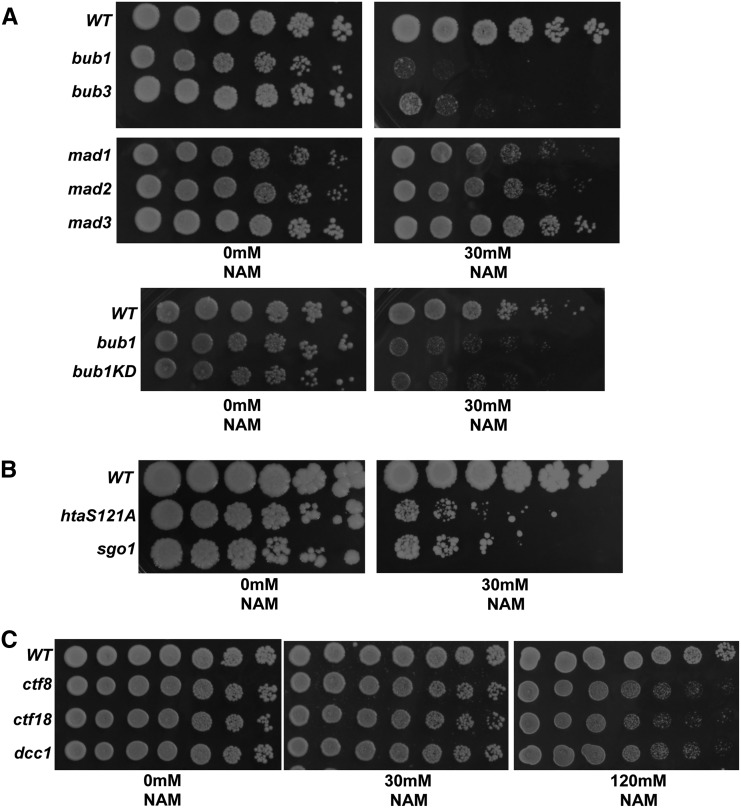
Growth assays show that mutants in the Bub1-Sgo1-H2A cohesion pathway render cells sensitive to NAM. (A) Deletion in *BUB1*, *BUB3*, and *BUB1* with its kinase domain deleted (*bub1KD*) all confer sensitivity to 30 mM nicotinamide (NAM). In contrast, deletions in *MAD1*, *MAD2*, or *MAD3* lead to little to no sensitivity to NAM. Loss of Bub1 kinase activity phenocopies the sensitivity displayed in *BUB1* deletion mutants to NAM. (B) The nonphosphorylatable *H2A* (*htaS121A*) mutant and deletion in *SGO1* both show similar sensitivity to NAM. (C) Deletions in subunits of the alternative replication complex leads to sensitivity to 120 mM NAM. Overnight cultures of each strain were serially diluted fivefold and 3 μl were spotted and incubated at 30°C. WT, wild-type.

Our screen identified deletions in *BUB1* and *BUB3* as highly sensitive to NAM (Table 1). Bub1p and Bub3p form part of the spindle assembly checkpoint (SAC) complex that is crucial in sensing a lack of microtubule-kinetochore attachments (London and Biggins 2014). In addition, Bub1 is required for the assembly of centromeric cohesion (Hoyt *et al.* 1991; Kawashima *et al.* 2010; Fernius and Hardwick 2007). Importantly, both functions are conserved from yeast to humans (Lara-Gonzalez *et al.* 2012). Thus, we sought to determine if the sensitivity to NAM observed in deletions of *BUB1* and *BUB3* is related to SAC and/or cohesion function. In addition to Bub1p and Bub3p, Mad1p, Mad2p, and Mad3p are required for SAC function (Li and Murray 1991; Hardwick and Murray 1995; Hardwick *et al.* 2000). If the defects conferred by NAM treatment required an intact SAC, we predicted that deletions in the *MAD* genes would also confer a similar sensitivity. Deletions in *MAD1*, *2*, and *3* were present in the library of deletions that we screened; however, these strains were not sensitive to NAM (Table S2 and Table S3). To rule out the possibility that these were false negatives, we performed growth assays using deletions in *MAD1*, *MAD2*, and *MAD3* (Figure 5A). Unlike *bub1*Δ and *bub3*Δ, which displayed sensitivity to NAM, *mad1*Δ, *mad2*Δ, and *mad3*Δ strains did not exhibit growth defects on NAM medium (Figure 5A and Figure S1). Therefore, the sensitivity of *bub1*∆ and *bub3*∆ to NAM may not be due to their role in SAC.

Bub1p in budding yeast, fission yeast, and humans is required for centromeric localization of Sgo1p, which is important for assembly of centromeric cohesion (Kawashima *et al.* 2010). In budding yeast, Bub1p phosphorylates H2A on serine 121 and this mediates recruitment of Sgo1p to the centromere. Thus, we sought to determine if loss of Bub1p kinase activity alone would cause NAM sensitivity. Indeed, we found that the kinase-deficient *bub1KD* mutant also showed growth sensitivity on NAM medium (Figure 5B). To test if NAM sensitivity was related to Bub1p’s function in cohesion, we tested deletions in *SGO1* and the nonphosphorylatable H2A mutant for sensitivity to NAM. Consistent with NAM having an effect on centromeric cohesion, we found that both *sgo1*Δ and the *htaS121A* strains were as sensitive to NAM as *bub1*Δ (Figure 5B). Taken together, these results suggest that the sensitivity of *bub1*∆ and *bub3*∆ to NAM is due to their role in cohesion and not to a defect in the SAC.

### Cohesin mutants are sensitive to NAM

**Figure 6:**
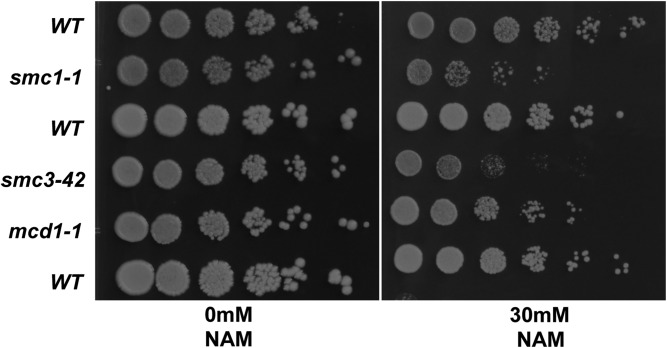
Growth assays reveal that conditional mutants in the essential cohesion complex subunits are sensitive to NAM. Strains with temperature sensitive mutations in the core cohesin genes (*smc1-1*, *smc3-42*, and *mcd1-1*) display sensitivity to nicotinamide (NAM) at permissive temperature. Overnight cultures of each strain were serially diluted fivefold and 3 μl were spotted and incubated at 30°C.

The sensitivity of *bub1*Δ, *sgo1*Δ, and *htaS121A* mutants suggests that defects in cohesion renders cells sensitive to NAM. In addition to *bub1*Δ and *bub3*Δ, deletions in all three subunits of the topoisomerase III complex (*TOP3*, *RMI1*, *SGS1*) were identified as highly sensitive (Figure 2 and Table 1). Notably, the Top3p complex plays an important role not only in resolving recombination intermediates and telomere stability, but also in cohesion assembly (Lai *et al.* 2007). Our primary screen also revealed that several subunits that comprise the alternative replication machinery, which functions in cohesion assembly, scored slightly higher than our 0.5 cut-off for sensitive mutants (Mayer *et al.* 2001)(Table S2 and Table S3). Hence, we tested a subset of mutants in the alternative replication complex and determined that deletions in *ctf8*, *ctf18*, and *dcc1* showed mild sensitivity to growth on plates containing 120 mM NAM (Figure 5C). The very mild NAM sensitivity of these strains may be due to redundancy in these pathways/genes. Together, these results support our conclusion that cohesion mutants are sensitive to NAM. To further explore the effect of NAM on cohesion, we examined NAM sensitivity of conditional alleles for essential cohesin genes (*SMC1*, *SMC3* and *MCD1*) that were not present in our genome-wide screen (Guacci *et al.* 1997; Michaelis *et al.* 1997). Consistent with cohesion defects leading to sensitivity to NAM, we found that *smc1-1* and *smc3-2* strains were highly sensitive to NAM and that the *mcd1-1* strain was mildly sensitive to NAM (Figure 6).

**Figure 7:**
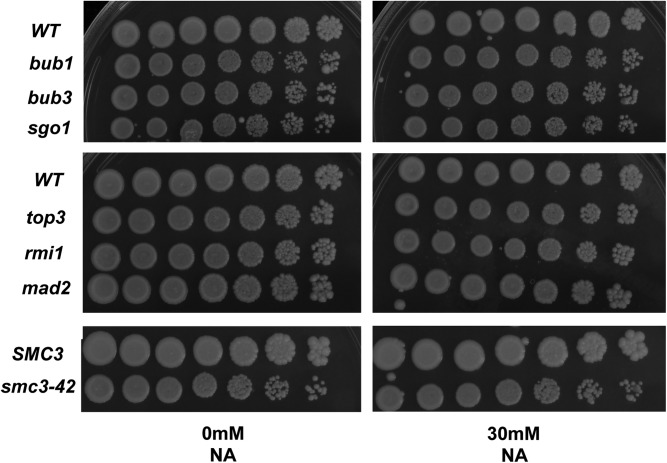
Growth assays of yeast carrying mutations in genes with defects in cohesion reveal an absence of sensitivity to nicotinic acid (NA). Each mutant tested here displays marked sensitivity to nicotinamide (NAM) but they are not sensitive to NA. Overnight cultures of each strain were serially diluted fivefold and 3 μl were spotted and incubated at 30°C.

The deacetylation reactions carried out by the sirtuins (Sir2p, Hst1p-4p) consume NAD^+^ yielding NAM and 2′O-acetyl ADP-ribose. NAM can be used in the “NAD^+^ salvage” pathway, first by conversion to NA by nicotinamidase (Pnc1p), followed by several enzymatic steps to yield more NAD^+^ (Bogan and Brenner 2008; Wierman and Smith 2014). Therefore, another possible explanation for the observed effects of NAM might be through the increased production of NA or potentially by increasing the levels of NAD^+^. We performed growth assays for several deletion and temperature sensitive mutants that affect cohesion in the presence or absence of 30 mM NA. As shown in (Figure 7) we observed no growth effects for any mutants tested on NA. These results support our conclusion that the effect of NAM is likely through its activity as an inhibitor of the sirtuins and perhaps an unknown activity of NAM that is independent of NAD^+^ biosynthesis.

### Summary

The results of our genome-wide screen show that genome stability pathways are particularly vulnerable to loss of sirtuin activity. A chemical-genomics approach using NAM, a pan-sirtuin inhibitor, provided novel insights into sirtuin-dependent activities in the cell, as demonstrated by the majority of genes we identified not previously being reported to have genetic interactions with sirtuin deletions. Importantly, GeneMANIA analysis of the 55 mutants revealed networks of genetic and physical interactions that have important functions in responding to and repairing DNA lesions. We also identified genes required for Golgi, vacuolar, and ribosome function, suggesting that sirtuin activity is indispensable for these processes. Gene deletions in these processes, which are not typically thought to be part of the DDR pathway, nonetheless have activities that relate to DDR directly or indirectly. For example, deletions in several Golgi genes (*YPT6*, *COG8*, *VPS53*) have negative interactions with DNA repair and recombination mutants. Many of the same genes that are important for DNA damage have been reported to have roles in cohesion assembly/maintenance (*e.g.*, *TOP3*, *HTZ1*). In turn, we showed that yeast deleted in *BUB1*, *BUB3*, *SGO1*, or carrying the H2A mutant that is nonphosphorylatable by Bub1 are all highly sensitive to NAM. In contrast, deletions in *MAD1*, *MAD2*, or *MAD3* did not result in NAM sensitivity. Taken together, these results indicate that the role of *BUB1* and *BUB3* in cohesion contributes to their sensitivity to NAM. In addition, mutants in genes that encode the essential cohesins are also sensitive to NAM, further showing that sirtuin activity is required when chromosome cohesion is compromised. Cohesion is known to have an important role in DNA damage repair and can form postreplicatively in response to DNA damage, supporting the role of sirtuins in protecting the genome. Moreover, this work confirms previous studies using NAM and deletions in the *HST3* and *HST4* sirtuins, which revealed important roles for *HST3*- and *HST4*-mediated H3K56 deacetylation in suppressing spontaneous DNA damage and establishing sister chromatid cohesion during S-phase (Maas *et al.* 2006; Celic *et al.* 2006, 2006; Thaminy *et al.* 2007). Our genome-wide screen for NAM sensitive mutants reveals biological pathways that are dependent on sirtuin activity, provides insights into the range of processes sirtuin activity impacts, and may aid in the identification of therapeutic targets for sirtuin-related diseases.

## Supplementary Material

Supporting Information
