# Supplementary material for: A Genome-Wide Screen with Nicotinamide to Identify Sirtuin-Dependent Pathways in Saccharomyces cerevisiae
Source: G3 (Bethesda). 2015 Dec 7;6(2):485–94. doi: 10.1534/g3.115.022244 (PMC4751566; doi:10.1534/g3.115.022244)
Supplement: Supporting Information [file supp_6_2_485__index.html]

A Genome-Wide Screen with Nicotinamide to Identify Sirtuin-Dependent Pathways in Saccharomyces cerevisiae — Supporting Information 

# A Genome-Wide Screen with Nicotinamide to Identify Sirtuin-Dependent Pathways in *Saccharomyces cerevisiae*

## Supporting Information for Choy *et al.*, 2016

**Files in this Data Supplement:**

- Figure S1 - Growth assays reveal that deletions in *MAD1*, *MAD2*, and *MAD3* are not sensitive to NAM. (.pdf, 131 KB)
- Table S1 - Scores and description of top 59 genes. (.xlsx, 21 KB)
- Table S2 - Normalized scores and ratios from Screen 1.(.xlsx, 302 KB)
- Table S3 - Normalized scores and ratios from Screen 2. (.xlsx, 313 KB)
- Table S4 - Genes identified in screen not previously reported to interact with sirtuins and their human orthologs. (.xlsx, 28 KB)
- Table S5 - GO Term Mapper Results. (.xlsx, 57 KB)
- Table S6 - Strain List. (.xlsx, 30 KB)
