# Supplementary material for: A Genome-Wide Screen with Nicotinamide to Identify Sirtuin-Dependent Pathways in Saccharomyces cerevisiae
Source: G3 (Bethesda). 2015 Dec 7;6(2):485–94. doi: 10.1534/g3.115.022244 (PMC4751566; doi:10.1534/g3.115.022244)
Supplement: Supporting Information [file supp_g3.115.022244_FigureS1.pdf]

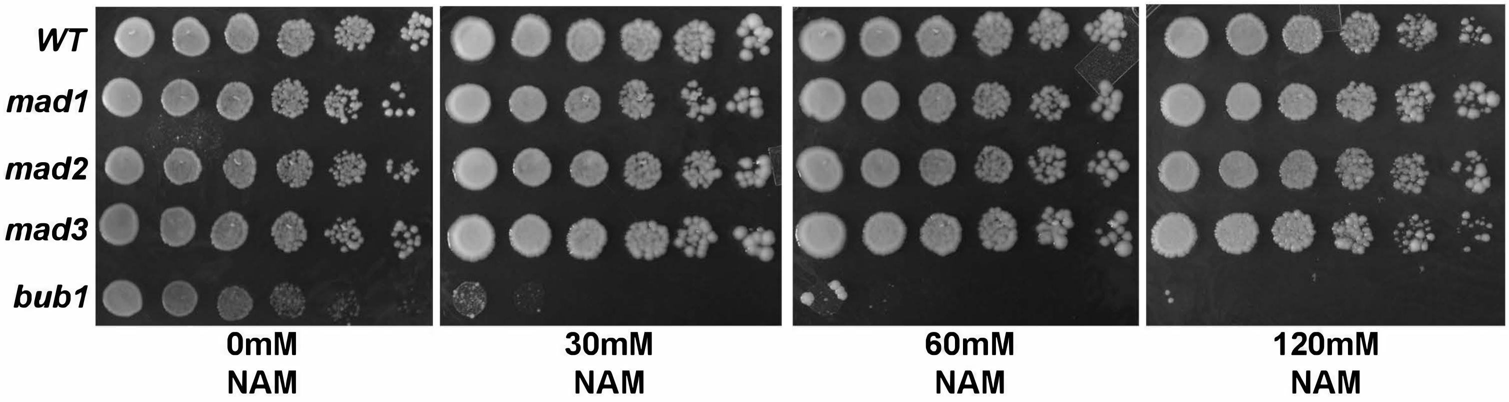

Figure S1 Growth assays reveal that deletions in *MAD1*, *MAD2*, and *MAD3* are not sensitive to NAM. We subjected each strain to the indicated concentrations of NAM and even at 120mM NAM, deletions in *MAD1*, *MAD2*, and *MAD3*, grew as well as wild-type (WT) cells. In contrast, deletion in *BUB1* displays a severe growth defect even at the lowest concentration of NAM (30mM). Overnight cultures of each strain were serially diluted five-fold and 3ul were spotted and incubated at 30°C. Plates were imaged at day 2 for 0mM NAM, day 4 for 30 and 60mM NAM, and day 7 for 120mM NAM.
